# Editorial: Photoactive Interfaces for Solar Energy Conversion

**DOI:** 10.3389/fchem.2019.00492

**Published:** 2019-07-10

**Authors:** Stefano Caramori, Marina Freitag, Mariachiara Pastore

**Affiliations:** ^1^Department of Chemical and Pharmaceutical Sciences, University of Ferrara, Ferrara, Italy; ^2^Department of Chemistry – Ångström Laboratory, Physical Chemistry, Uppsala University, Uppsala, Sweden; ^3^Laboratoire de Physique et Chimie Théoriques, Université de Lorraine and CNRS, Nancy, France

**Keywords:** solar energy, dye-sensitized solar cells, DFT, TiO_2_, p-type semiconductor

A path toward a successful solar-driven technology is via construction of durable, efficient, and cost-competitive solar cells, which are capable of effectively exploiting sunlight to produce electricity or fuels. To this end, the fundamental understanding and detailed characterization of heterogeneous photoactive interfaces aimed toward sun harvesting is crucial to boost the device performances. This field is growing and evolving very rapidly and is the subject of increasing research efforts. The challenge stems from the structural complexity of the interfaces and of the related physico-chemical phenomena, whose interlacing determines the overall efficiency of the cells. Indeed, the prerequisites for broad spectral harvesting and favorable energy-level alignment for the intended solar-driven process should be coupled with fast charge separation and collection, competing successfully with photogenerated charge recombination. Efficiency should also come with material and device robustness and stability under demanding operational conditions (i.e., humidity, heat, oxygen, extreme pH conditions in aqueous media), particularly in the case of photoelectrosynthetic cells for solar fuel production. To this end, the understanding and the accurate theoretical prediction of surface energetics is crucial to design and employ new material and processes and to rationally optimize photoactive interfaces.

The contribution by Kim et al. explores the electronic structure of different crystalline phases of titania using a newly developed self-consistent optimization approach of hybrid functionals and DFT+U methods, finding that this computationally efficient method delivers predictions on structural and electronic properties of TiO_2_ phases which are comparable with the experimental and higher level GW results. This methodology is further applied to rutile and anatase interfaces, extremely popular photocatalysts for energy and environmental applications, showing that the solvent medium has a strong influence in band alignment, resulting in almost isoenergetic conduction band minima of these two phases. The transition from the straddling-type to the staggered-type band alignment between rutile and anatase phases could be thus tuned by the choice of the electrolyte medium.

As far as interfacial complexity is concerned, the review article by Boschloo discusses possible ways of improving dye-sensitized solar cells (DSCs), where molecular design of dye sensitizers can lead to endless modifications for optimizing solar performances. Key issues will be the achievement of optimal dye packing on the TiO_2_ surface to increase light absorption and to achieve a better blocking effect to minimize recombination. Co-sensitization, where two or more different dyes are co-loaded on the semiconductor surface, offers good possibilities in this respect. These dyes shall be coupled to novel redox mediators and hole transporting materials (HTMs) capable to offer much higher output voltage than the traditional triiodide/iodide redox couple. High-performance DSCs are indeed of interest for many applications, ranging from power source for consumer electronics, to building integrated PV and large-scale power generation. The option of high transparency in the near infrared region also opens up for the use of DSC as a top cell in tandem solar cells device.

In line with the research lines outlined by Boschloo, the research article by Bonomo et al. explores indeed interfacial effects with squaraine dyes, red-infrared absorbing dyes applied to p-type DSCs based on NiO, which could be used to increase photovoltage through coupling with n-type photoanodes. The authors combine experimental design approach and electrochemical impedance spectroscopy (EIS) investigations to optimize the cell performances, finding that NaOH treatment passivates Ni^3+^ defective sites, avoiding hole trapping and recombination and thus leading to higher current densities and better fill factor. Also in this case the combination of optimal dye packing with surface pre-treatment is exploited for maximized power output.

Muñoz-Garcia et al. reported the first theoretical study of CuGaO_2_ delafossite with Mg doping and its adsorption properties toward two anchoring groups as an alternative to NiO for p-type DSCs. The authors were able to show high selectivity with the *ab-initio* studies toward monodendate and Ga surface binding and further indicating, that despite the p-type metal oxide difficult synthesis, it might deliver higher performance than NiO through higher sensitization.

Current strong trend in hybrid solar cells, DSCs and perovskite solar cells toward their application in diffuse light conditions was cemented by the contribution of Juang et al. The work demonstrated highly efficient energy harvesting under low light conditions of 200 lux. In focus was the use of cobalt based redox mediators in DSCs with organic sensitizer MK-2, with PCE of 16% surpassing the efficiency of Iodine based electrolytes. The authors pointed out that the results showed the importance to reduce the potential loss for further improvement of DSCs, especially for indoor application.

## Author Contributions

All authors listed have made a substantial, direct and intellectual contribution to the work, and approved it for publication.

### Conflict of Interest Statement

The authors declare that the research was conducted in the absence of any commercial or financial relationships that could be construed as a potential conflict of interest.

